# Postpartum Mental Health Problems in the Mothers of Very, Moderately and Late Preterm Infants During and After COVID–19 Pandemic: A Cross‐Sectional Comparison Study and Possible Risk Factors

**DOI:** 10.1155/da/5236584

**Published:** 2026-05-14

**Authors:** Magdalena Chrzan-Dętkoś, Natalia Murawska, Marta Łockiewicz

**Affiliations:** ^1^ Institute of Psychology, University of Gdańsk, Gdańsk, Poland, ug.edu.pl

**Keywords:** COVID-19 pandemic, mental health problems, postpartum depression, preterm birth, risk factors

## Abstract

**Background:**

Preterm birth (PTB) affects ~11% of women worldwide. The COVID‐19 pandemic put an additional burden on this already challenging situation.

**Aims:**

We aimed to analyse (1) the impact of the COVID‐19 pandemic on the severity of specific depressive, anhedonia, anxiety and self‐harm thoughts (SHTs) symptoms in mothers of preterm babies, (2) differences in the severity of these symptoms among mothers who gave birth very preterm, moderately and late preterm and (3) potential socio‐demographic, psychological, birth‐related and COVID‐19‐related factors associated with an increase in the mothers’ scores.

**Methods:**

A total of 948 mothers of preterm children took part in a midwife‐led postpartum depression (PPD) screening; 543 of them gave birth during the pandemic, and 405—after. A sociodemographic survey and the Edinburgh postnatal depression scale (EPDS) were administered. To investigate possible differences between comparison groups, we conducted *t*‐tests, one‐way ANOVAs with Tukey post hoc tests, and chi‐square tests. Several hierarchical multiple regression analyses were performed to assess possible risk factors for postpartum mental health problems.

**Results:**

We found that mothers who gave birth to very preterm children scored higher in depressive symptoms than mothers of moderately and late preterm children. In general, women who gave birth during COVID‐19 scored higher in anxiety (a statistical trend), but not in depression. The significant predictors of increased mental health problems were a worse financial situation, a higher education level, previous maternal mental health problems and a child’s physical health (lower birth weight and the presence of medical complications during delivery).

**Conclusions:**

The severity of depressive symptoms was not explained by COVID‐19, but gestational age. It shows that mothers of very preterm infants need additional attention and support in the postpartum period.

## 1. Introduction

Preterm birth (PTB), a leading cause of death in children under the age of 5 [1], affects ~11% of women worldwide [2]. In Poland, as in other European high‐income countries, the prevalence of PTB has remained stable in the last years (2020–2023) at ~7.2%–7.4% of all births [1, 3].

Many preterm children often experience long‐term disabilities, such as physical impairments or neurodevelopmental disorders [1]. Babies born extremely preterm (defined as gestational age less than 28 weeks) face the highest risk of mortality and morbidity, then those born very preterm (between 28 and less than 32 weeks), followed by children born moderate to late preterm (32 weeks to less than 37 weeks) [4, 5].

PTB may affect maternal mental health through multiple mechanisms. A child’s admission to the neonatal intensive care unit (NICU), due to PBT, negatively impacts the mothers’ transition to motherhood, due to, among others, a feeling of disconnection with the baby [6]. As in NICU infants and parents are separated, both physically and emotionally, though to a different degree, parents experience adverse mental health outcomes (e.g., depressive symptoms) and fulfil their parental roles less efficiently (even to the point of withdrawal) [7]. As they cannot prepare themselves for PTB, they commonly struggle with emotional shock, fear, symptoms of anxiety, depression and post‐traumatic stress [8–10]. de Paula Eduardo et al.’s [11] systematic review and meta‐analysis identified PTB as a risk factor for postpartum depression (PPD). Moreover, a systematic review by Malouf et al. [12] estimated that, at 1 month after birth, the prevalence rates of anxiety and post‐traumatic stress among parents with infants admitted to NICU were as high as 41.9% and 39.9%, respectively. This is twice as high as the prevalence reported in Wang et al.’s [13] study, which aimed to assess the global epidemiology of PPD. In Poland, in midwife—led screening, a higher risk of PPD is observed in 6.2% of postpartum mothers [14]. Even when the clinical thresholds of PPD or anxiety are not met, parents frequently struggle with hypervigilance and panic, through the NICU stay to the transition home [10, 15]. As many as 34% of mothers with hospitalised preterm infants experience suicidal thoughts, compared with 14% term children’s mothers [16]. PBT is described as a sudden and unexpected event that triggers extreme stress, feelings of disorientation, fear for the child’s health and life and a sense of loss of control or helplessness [17, 18]. Such experiences have been linked to postpartum psychological distress [19]. PBT may disrupt the natural trajectory of hormonal adaptation associated with the sudden withdrawal of reproductive hormones and the heightened activation of stress‐related systems (e.g., elevated cortisol levels), which may contribute to stress‐related dysregulation, an increased vulnerability and the development of postpartum mood disorders [20, 21].

Even though the literature consistently shows adverse mental health outcomes in mothers of preterm infants, their contributing factors are less clear. The investigations are complicated by the fact that many risk factors for PPD are also linked to premature births. Specifically, depression during pregnancy, socio‐economic status (SES), social support, stress, antenatal anxiety and stressful life events influence both the timing of delivery [22, 23] and the incidence of PPD [24, 25]. Moreover, Yaari et al. [26] reported that the higher family social risk was associated with greater distress among mothers of very preterm infants. A very low birthweight posed a 4–18 higher risk for PPD, more relevant than a lifetime psychiatric disorder, low social support and SES [27]. Other studies also found that mothers of moderate and late preterm infants were at increased risk of PPD symptoms. A systematic review by Vigod et al. [28] showed that for such mothers the rates of PPD were as high as 40% during the first 12 weeks postpartum; these elevated scores reflected; however, a transient distress rather than PPD. A sustained depression was linked with the degree of prematurity, lower birth weight, persistent infant illness/disability and self‐assessed lack of social support [28]. Similarly, Yaari et al. [26] reported that mothers of preterm infants, as compared with mothers of term infants, manifested higher psychological distress, even several years after a child’s birth, which worsened over time in case of a higher neonatal medical risk.

The COVID‐19 pandemic could, potentially, have been another risk factor for the increase of PPD and anxiety symptoms in mothers of preterm‐born children, as it put an additional burden on the parents. COVID‐19 led to a worldwide reduction in parents’ access to NICUs and, consequently, their involvement in infant care. When the pandemic started in March 2020, most NICUs implemented state—mandated restrictions, including the prohibition of parental visits (although hospital policies varied across countries), which contributed to more frequent infant–parent separation [29]. Subsequently, among all postpartum mothers, the rate of depressive and anxiety symptoms increased significantly [30–32].

However, the findings of a few previous studies investigating a link between the pandemic and an increase in PPD in mothers of preterm born babies are contradictory. Comparing the time before and during the pandemic, the studies showed no increase in depression symptoms (Italy, [33]; Chile, [34]), a non‐statistically significant trend of a general increase (Switzerland, [35]), a higher proportion of mothers experiencing high levels of paediatric medical traumatic stress symptoms (PMTS; including intrusion, avoidance and having a clinical total score of PMTS) (Italy, [36]), and a higher proportion of mothers exceeding the cut‐off for possible clinical depression: 12% vs. 26%, but only for mothers of very and extremely preterm children (Belgium, [37]) or women in a disadvantaged social situation (Taiwan, [38]). Similarly, either no difference in stress [34] or a slight increase in parenting stress was observed (Italy, [33]). Moreover, during the second lockdown, it was the mothers of full‐term, as compared with of preterm children, who reported more depressive symptoms (Germany [39],). Itoshima et al. [40] found no link between postpartum depressive symptoms in mothers of preterm infants in NICUs and COVID‐19 restriction measures.

Some of these discrepancies could be explained by the aforementioned differences in the restrictions of parental visits in NICUs between countries. In Polish hospitals, including those in northern Poland, where the study took place, parents were prohibited from NICU visits in the first year of the pandemic, before the implementation of the vaccinations: in March 2020, national guidelines issued by the Chief Sanitary Inspector restricted hospital visits without exceptions for parents [41]. This resulted in lower levels of caregiving capacities among parents of hospitalised newborns, lower caregiving self‑efficacy, poorer preparation for post‑discharge care and reduced caregiving outcomes related to speech therapy recommendations after discharge [42]. In other countries, the NICU parental visits were restricted, but depending on the hospital policy, still possible [29, 43] or replaced by video visits [38], which was associated with a significant depressive and anxiety symptom reduction. Moreover, after discharge from a hospital, parents could have felt unsupported with the care of their preterm‐born babies, often having special care needs, as NICUs sometimes reduced therapy services, lactation medicine and/or social work support (43% of those surveyed in [43], in the USA; [29]).

In Poland, the post‐discharge care for preterm infants is described in the *Standards of Ambulatory Care for Preterm Infants* developed by the Polish Neurological Society and the Polish Paediatric Society [44]. However, during the COVID‐19 pandemic, specialist care, including visits to neurologists, neonatologists, ophthalmologists, psychologists and physiotherapists, was provided either online or with severe limitations [45]. Standard home visits by community midwives were also substantially restricted or replaced by telehealth [45].

In Poland, since 2019, midwives have been obliged to screen for PPD risk and symptoms twice during pregnancy and once in the postpartum [46]. During the pandemic, this screening was often offered via online platforms, without opportunities for discussion or immediate support from the midwife. Despite the increased prevalence of mothers at risk of PPD observed during the first year of the COVID‐19 pandemic, referral rates remained low, with only about 8% of potentially affected mothers following referral advice [30, 47]. These changes in the organisation of perinatal and newborn care could have exacerbated the parents’ stress and increased the mental health problems risk. Restrictions or suspension of parental visits due to the epidemic state also deprived parents and infants of kangaroo care, which, to a moderate degree, protects the mothers from the negative effects of PTB and low birth weight [48].

As maternal PPD symptoms partially mediate and aggravate the association between PTB and child mental disorders, timely prevention interventions are crucial [49]. Thus, we hypothesised that the restrictions and either a lack of or changes in the standard care and institutional support in PTB situations could result in an increased prevalence of mental health problems among mothers of preterm children. No other studies investigated the mental health outcomes, and their predictors, of Polish mothers of preterm infants during the COVID‐19 pandemic. Moreover, as none of the studies discussed above compared the pre‐ and postpandemic PPD prevalence rates (the comparisons were of pre‐ and during pandemic rates), our study, to the best of our knowledge, is the first one to do so.

Based on the literature, we formulated the following hypotheses:

1. The rate of depressive, anhedonia, anxiety and self‐harm thoughts (SHTs) symptoms would be higher in mothers of preterm children born during the COVID‐19 pandemic than in mothers of preterm children born after its end.
2. The rate of depressive, anhedonia, anxiety and SHT symptoms would be highest for mothers who gave birth very preterm, as compared with mothers who gave birth moderately preterm and late preterm.
3. The socio‐demographic (including age, education, self‐assessed financial situation and relationship status), psychological (including previous mood disorders), birth‐related (including the degree of prematurity, type of birth, type of pregnancy, a child’s condition after birth and the presence of complications at birth in a child’s health) and COVID‐19‐epidemic‐state factors would be associated with an increased depressive, anhedonia, anxiety and SHTs score among mothers of preterm children.

## 2. Method

### 2.1. Study Design

In our analyses, we utilised data gathered during the realisation of the *Next Stop: Mum* PPD prevention programme implemented by the Polish Ministry of Health, covering the northern region of Poland. The project was overseen by a consortium formed by the *Copernicus Podmiot Leczniczy* sp. *z o. o*. and the *University of Gdańsk* with co‐financing by the EU Society Funds under the Operational Programme: Knowledge Education Development POWR.05.01.00‐IP.05‐00‐006/18. The data were collected through screenings conducted directly by midwives during the first year postpartum (*M* = 1.32 months, SD = 2.15, Min. = 0, Max. = 11 months). Midwives collected data from all new mothers during their stay in hospitals’ maternity wards after childbirth, during routine postnatal home visits, immunisation appointments or standard medical check‐ups. Participants provided written consent to take part in the study. All collected data were securely entered into the database by psychologists and trained psychology graduate students.

The study was conducted in accordance with the Declaration of Helsinki. The Ethics Board for Research Projects of the *University of Gdańsk* had approved the study protocol. The programme ended on September 30, 2023, but the data are still being processed. Thus, the database used in the present study partly overlaps with the databases used in other studies^1^. The present study focuses on the relationship of PTB and other risk factors with maternal mental health outcomes (depressive, anxiety and anhedonia symptoms and SHTs) during and after the COVID‐19 pandemic.

In this study we analysed data from all eligible mothers who had met the predefined inclusion criteria (PTB during and after the COVID‐19 pandemic) selected from the project’s database. The degree of prematurity was defined based on the gestational age extremely and very preterm (≤32 weeks), moderate preterm (33 to ≤34 weeks), and late preterm (35 to ≤36 weeks), following the World Health Organisation (WHO) categories [50]. The COVID‐19 pandemic was defined as the period between March 20, 2020, and May 16, 2022 (when an epidemic state was in force in Poland—the Regulation of the Minister of Health [51]; Regulation of the Council of Ministers [52]). We excluded mothers of full‐term infants and mothers who gave birth before the COVID‐19 pandemic. Due to the use of routinely collected data as a part of a health prevention programme, a sample size calculation was not performed.

### 2.2. Measures

• Socio‐demographic and health data survey: developed by the first author. It includes information about a woman’s SES, maternal marital status, place of residence, her and her child’s health peripartum, including birth weight, gestational age, number of days in the NICU and a child’s health complications. The data were gathered by a midwife during a semi‐structured interview, based on the questions presented in Supporting information.
• The Edinburgh postnatal depression scale (EPDS): EPDS is an instrument assessing depressive symptoms in mothers in the peripartum period, translated to over 60 languages [53, 54]. It contains 10 items, each scored from 0 to 3 points. A higher score indicates more severe depressive symptoms. The mean total score of EPDS was used as the primary outcome of this study. Additionally, as a secondary outcome, the incidence of a possible clinical depression was estimated using a cutoff score of ≥13 points, which identifies women with clinically significant levels of depressive symptoms (referred to as a clinical score) [55]. We chose this threshold, as prior systematic reviews and meta‐analyses had indicated that it identifies clinically significant depressive symptoms during pregnancy and postpartum with a higher specificity [55, 56]. In our sample, Cronbach’s alpha was 0.882.
• EPDS 3A for anxiety: a composite score of items 3, 4 and 5 assesses anxiety symptoms. The maximum score is 9 points. The mean total score of EPDS 3A was used as the primary outcome. Additionally, as a secondary outcome, the incidence of possible clinical anxiety was estimated using the cutoff score of ≥6 points (a clinical score) [57]. The Cronbach’s alpha for the subscale was 0.857 [57] (in our sample, the Cronbach’s alpha for EPDS 3A was lower, but still on an acceptable level, at 0.788). This version was previously used in research on PPD, for example, by Pan and Xu [58].
• EPDS for anhedonia: a composite score of items: 1 and 2 assesses anhedonia symptoms [59]. The maximum score is 6 points. The mean total score was used as the outcome. In our sample, Cronbach’s alpha was 0.760. This subscale was previously used in research on PPD, for example, by Giliberti et al. [60] and Zanardo et al. [61].
• EPDS item 10: assesses the prevalence of SHTs. The maximum score is 3 points. The mean total score of this item was used as the outcome. Women with a score ≥1 point were classified as experiencing SHTs (a clinical score). Women with a score = 0 were classified as not presenting SHTs. Here, we followed the approach in Wisner et al. [62] and Iliadis et al. [63].

### 2.3. Participants

A total of 948 mothers of prematurely born children participated in our study. Of those, 543 gave birth during the pandemic, and 405—after. In the entire group, 490 mothers gave birth to late preterm babies (231 after the pandemic and 259 during the pandemic), 202 to moderate preterm babies (88 after and 114 during), and 256 to very preterm babies (86 after and 170 during). The mean age was 30.94 years (SD = 5.68, range: 16–47), the mean gestational age was 33.69 weeks (SD = 2.71). Mothers who gave birth during the pandemic (*M*_age_ = 30.74 years, SD = 5.62, range: 16–46) were of the same age as mothers who gave birth after the pandemic (*M*_age_ = 31.21 years, *SD* = 5.75, range: 19–47; *t*(918) = 1.25, *p* = 0.106). However, the mean gestational age for mothers who gave birth during the pandemic (*M* = 33.39 weeks, SD = 2.93) was lower than that for those who gave birth after the pandemic (*M* = 34.10 weeks, SD = 2.32, *t*(942.732) = 4.12, *p* = 0.001). A detailed sociodemographic characteristic of the sample is presented in Table 1. Women assessed during the COVID‐19 pandemic were more likely to have had a single low‐risk pregnancy. The condition of their babies was less often described as good and more often as asphyxia. These mothers also lived in smaller urban areas, as compared with those assessed after the COVID‐19 pandemic.

**Table 1:** Descriptive statistics.

Baseline characteristic	Preterm birth			Statistics	
	All women	During the pandemic	After the pandemic		
	*n* (%)	*n* (%)	*n* (%)	*p*	*χ* ^2^ (*df*)
Type of pregnancy					
Single	907 (95.7%)	543 (100%)	364 (89.9%)	<0.001 ^∗∗^	57.46 (1)
Type of pregnancy					
At‐risk	469 (50.8%)	252 (47.5%)	217 (55.2%)	0.020 ^∗^	5.44 (1)
Low‐risk	455 (49.0%)	279 (52.5%)	176 (44.8%)		
No data	24 (2.5%)	12 (2.2%)	12 (3.0%)		
Child’s condition after birth					
Good	704 (79.2%)	389 (76.3%)	315 (83.1%)	0.018 ^∗^	10.03 (3)
Hypoxia	127 (14.3%)	77 (15.1%)	50 (13.2%)		
Asphyxia	48 (5.4%)	36 (7.1%)	12 (3.2%)		
No signs of life	10 (1.1%)	8 (1.6%)	2 (0.5%)		
No data	59 (6.2%)	33 (6.1%)	26 (6.4%)		
Type of birth					
C‐sections	507 (53.7%)	276 (51.2%)	231 (43.0%)	0.075^a^	3.16 (1)
Vaginal birth	437 (46.3%)	263 (48.8%)	174 (57.0%)		
No data	4 (0.4%)	4 (0.7%)	—		
Marital status					
Married	620 (65.5%)	346 (63.7%)	274 (68.0%)	0.415	2.85 (3)
Partnered	305 (32.2%)	183 (33.7%)	122 (30.3%)		
Single parent	16 (1.7%)	10 (1.8%)	6 (1.5%)		
Other	11 (0.6%)	4 (0.7%)	1 (0.2%)		
No data	2 (0.2%)	—	2 (0.5%)		
Education					
Postgraduate higher education degree	461 (48.7%)	263 (48.4%)	403 (49.1%)	0.739	1.98 (4)
Graduated from a secondary school	312 (33.0%)	173 (31.9%)	139 (34.5%)		
Vocational certificate	102 (10.8%)	63 (11.6%)	39 (9.7%)		
Lower secondary school	32 (4.0%)	23 (4.2%)	15 (3.7%)		
Basic education	33 (3.5%)	21 (3.9%)	12 (3.0%)		
No data	2 (0.2%)	—	2 (0.5%)		
Financial situation					
Very good	184 (19.7%)	96 (17.9%)	88 (22.1%)	0.101	7.77 (4)
Good	637 (68.3%)	363 (67.9%)	274 (68.8%)		
Average	96 (10.3%)	64 (12.0%)	32 (8.0%)		
Bad	10 (1.1%)	7 (1.3%)	3 (0.8%)		
Very bad	6 (0.6%)	5 (0.9%)	1 (0.3%)		
No data	15 (1.6%)	8 (1.5%)	7 (1.7%)		
Place of residence					
Urban area: more than 250,000 residents	324 (34.8%)	190 (35.4%)	134 (33.9%)	0.005 ^∗∗^	15.03 (4)
Urban area: up to 250,000 residents	61 (6.5%)	38 (7.1%)	23 (5.8%)		
Urban area: up to 100,000 residents	93 (10%)	67 (12.5%)	26 (6.6%)		
Urban area: up to 50,000 residents	153 (16.4%)	73 (13.6%)	80 (20.3%)		
Rural areas	301 (32.3%)	169 (31.5%)	132 (33.4%)		
No data	16 (1.7%)	6 (1.1%)	10 (2.5%)		
Disorder diagnosed in the past^b^					
Depression	75 (8.1%)	36 (6.8%)	39 (10.0%)	0.225	3.63 (3)
Bipolar affective disorder	1 (0.1%)	1 (0.2%)	—		
Other mood disorders	31 (3.4%)	20 (3.8%)	11 (2.8%)		
None of the above	814 (88.4%)	475 (89.2%)	339 (87.1%)		
No data	27 (2.8%)	11 (2.0%)	16 (4.0%)		

^a^Statistical trend.

^b^The percentages do not add up to 100.00%, as the respondents could give more than one response. Other mood disorders denotes mood disorders different than depression or bipolar affective disorder.

^∗^ *p* ≤ 0.05.

^∗∗^ *p* ≤ 0.01.

### 2.4. Statistical Analysis

We used SPSS Version 29.0 for statistical analyses. Since our sample sizes were large, we used parametric tests following the central limit theorem [64]. Descriptive and frequency analyses were conducted to assess the distribution of sociodemographic characteristics by our respondents. To calculate the differences between comparison groups in postpartum disorder symptoms we carried out *t*‐tests, one‐way ANOVAs with Tukey post hoc tests, and chi‐square tests. Several hierarchical multiple regression analyses were performed. The degree of prematurity was entered as an independent variable in Step 1. The delivery during or after the COVID‐19 state of epidemic emergency was added as an independent variable in Step 2. Selected socio‐demographic factors (age, financial situation, education and marital status), maternal mental health prior to pregnancy, a child’s physical health (condition after birth and presence of medical complications), childbirth‐related factors (type of birth and birth weight) were entered as independent variables in Step 3. Depression, anxiety, anhedonia and SHTs’ severity symptoms were entered as dependent variables. The selection of the potential predictor variables for regression was decided based on the conducted literature review.

## 3. Results

### 3.1. The Severity and Prevalence of Depression, Anxiety, Anhedonia and SHTs Symptoms in Mothers of Preterm Infants During and After the COVID‐19 State of Epidemic Emergency

Descriptive statistics for the depression, anxiety, anhedonia and SHTs symptoms in mothers of preterm infants during and after the pandemic are presented in Table 2. We found no statistically significant differences in the mean levels of PPD, anhedonia and SHTs between groups of women who gave birth during and after the pandemic. Mothers who delivered their babies during the epidemic state, as compared with those who gave birth after it, scored higher in anxiety (a statistical trend).

**Table 2:** The depression, anxiety, anhedonia and SHTs symptoms in mothers of preterm infants during and after the pandemic—comparison between the groups.

Outcomes			*Statistics*		*Cohen’s d*
	*M*	SD	*t*(946)	*p*	
EPDS					
During the pandemic	5.13	4.69	1.07	0.287	0.07
After the pandemic	4.79	4.99			
Total	4.98	4.82			
EPDS anxiety					
During the pandemic	2.68	1.95	1.79	0.073^*^	0.12
After the pandemic	2.43	2.11			
Total	2.57	2.11			
EPDS anhedonia					
During the pandemic	0.55	0.77	0.18	0.861	0.01
After the pandemic	0.54	1.05			
Total	0.55	1.02			
SHTs					
During the pandemic	0.04	0.26	0.71	0.480	−0.05
After the pandemic	0.05	0.35			
Total	0.05	0.30			

^∗^ *p* < 0.10.

A chi‐square test was conducted to examine the association between the delivery, either during or after the COVID‐19 pandemic and depressive, anxiety and SHTs scores based on cut‐off points. The analysis revealed no significant associations between these variables (Table 3).

**Table 3:** Group differences for depression, anxiety and SHTs based on cut‐off points in mothers of preterm infants who gave birth either during or after the COVID‐19 pandemic.

Outcomes		Birth and COVID‐19 pandemic			Statistics	
		During	After	Total	*χ² (df)*	*p*
EPDS						
Above clinical cut‐off score	Count	38_a_	33_a_	71	0.44 (3)	0.506
	%	7.0%	8.1%	7.5%		
EPDS anxiety						
Above clinical cut‐off score	Count	56_a_	42_a_	98	0.00 (3)	0.977
	%	10.3%	10.4%	10.3%		
SHTs						
Above clinical cut‐off score	Count	17_a_	12_a_	29	0.02 (3)	0.882
	%	3.1%	3.0%	3.1%		

*Note:* Each subscript letter denotes a subset of COVID‐19 categories whose column proportions do not differ significantly from each other at the 0.05 level. A clinical score for EPSD was ≥13 points, for anxiety ≥6 points, for SHTs ≥1 point.

### 3.2. The Severity and Prevalence of Depressive, Anhedonia, Anxiety, and SHTs Symptoms in Mothers who Gave Birth Very Preterm, Moderate Preterm and Late Preterm Both During and After the Epidemic State

To investigate if the severity of depressive, anhedonia, anxiety and SHTs symptoms would be different for mothers who gave birth very preterm, moderate preterm and late preterm both during and after the epidemic state we calculated one‐way ANOVAs (Table 4). The Tukey post hoc test for multiple comparisons demonstrated that mothers who gave birth to very preterm children scored higher in depressive symptoms than mothers of moderate and late preterm children (a, statistical trend: *p* = 0.064 and 0.078, respectively). We found no group differences for anxiety, anhedonia and SHTs.

**Table 4:** The comparison of the severity of depressive, anhedonia, anxiety and SHTs symptoms in mothers who gave birth very preterm, moderate preterm and late preterm both during and after the epidemic state.

Outcomes	*M*	SD	Min.	Max.	*F* (*df*)	*p*	*η* ^2^
EPDS							
Very PTB	5.61	5.07	0	30	3.17 (2)	0.042	0.007
Moderate PTB	4.59	4.72	0	23	—	—	—
Late PTB	4.81	4.71	0	29	—	—	—
Total	4.98	4.82	0	30	—	—	—
EPDS anxiety							
Very PTB	2.76	2.10	0	9	1.89 (2)	0.152	0.004
Moderate PTB	2.38	2.21	0	9	—	—	—
Late PTB	2.56	2.06	0	9	—	—	—
Total	2.57	2.11	0	9	—	—	—
EPDS anhedonia							
Very PTB	0.64	1.15	0	6	1.60 (2)	0.202	0.003
Moderate PTB	0.48	0.93	0	5	—	—	—
Late PTB	0.53	0.99	0	6	—	—	—
Total	0.55	1.02	0	6	—	—	—
SHTs							
Very PTB	0.05	0.35	0	3	0.68 (2)	0.505	0.003
Moderate PTB	0.02	0.16	0	1	—	—	—
Late PTB	0.05	0.32	0	3	—	—	—
Total	0.05	0.30	0	3	—	—	—

Abbreviation: PTB, preterm birth.

A chi‐square test was conducted to examine the association between depressive, anxiety and SHTs symptoms based on cut‐off points in mothers who gave birth very preterm, moderate preterm and late preterm both during and after the epidemic state. The analysis revealed no significant association between these variables (Table 5).

**Table 5:** The comparison of the severity of depressive, anhedonia, anxiety and SHTs symptoms based on cut‐off points in mothers who gave birth very preterm, moderate preterm and late preterm both during and after the epidemic state.

Outcomes		Preterm Birth			Total	Statistics	
		Very	Moderate	Late		*χ² (df)*	*p*
EPDS							
Above clinical cut‐off score	Count	18_a_	15_a_	38_a_	71	0.13 (2)	0.938
	%	7.0%	7.4%	7.8%	7.5%		
EPDS ANXIETY							
Above clinical cut‐off score	Count	31_a_	19_a_	48_a_	98	1.21 (2)	0.546
	%	12.1%	9.4%	9.8%	10.3%		
SHTs							
Above clinical cut‐off score	Count	7_a_	5_a_	17_a_	29	0.60 (2)	0.740
	%	2.7%	2.5%	3.5%	3.1%		

*Note:* Each subscript letter denotes a subset of preterm birth category whose column proportions do not differ significantly from each other at the 0.05 level. Above clinical cut‐off score for EPSD was ≥13 points, for anxiety ≥6 points, for SHTs ≥1 point.

### 3.3. The Models of Relations Between the Degree of Prematurity, Giving Birth During or After the COVID‐19 Pandemic and Selected Aspects of Socio‐Demographic Factors, Maternal Mental Health Prior to Pregnancy, a Child’s Physical Health, Childbirth Related Factors and Depression, Anxiety, Anhedonia and SHTs Symptoms

The regression analysis (Table 6) for depression symptoms showed that the independent variables explained a total of 16.2% of the variance (*F*_(13, 782)_ = 11.64, *p*  < 0.001). Significant independent variables in Step 3 were financial situation, education, previous maternal mental health (showing that mothers with worse financial situation, higher educational level, with a prior diagnosis of a mood disorder scored higher in depressive symptoms), and a child’s physical health (showing that mothers of children born with medical complications and a lower birth weight scored higher in depressive symptoms).

**Table 6:** Results of hierarchical regression analyses in which the degree of prematurity, giving birth during or after the COVID‐19 pandemic and selected aspects of socio‐demographic factors, maternal mental health prior to pregnancy, a child’s physical health and childbirth related factors were regressed upon depression symptoms .

Step	Predictor variable		Depression symptoms		
			Beta	*t*	*p*
1	Preterm group	Moderate (ref. very)	−0.115	−2.708	0.007 ^∗∗^
		Late (ref. very)	−0.114	−2.670	0.008 ^∗∗^
*R* ^2^	0 .012	△*F* (*df*1, *df*2)	4.645 ^∗^ (2, 793)		
2	Preterm group	Moderate (ref. very)	−0.111	−2.616	0.009 ^∗∗^
		Late (ref. very)	−0.108	−2.538	0.011 ^∗^
	Birth during the COVID‐19 state of epidemic emergency		0.044	1.128	0.220
*R* ^2^	0 .013	△*F* (*df*1, *df*2)	1.509 (1, 792)	—	—
3	Preterm group	Moderate (ref. very)	−0.023	−0.544	0.587
		Late (ref. very)	0.043	0.830	0.407
	Birth during the COVID‐19 state of epidemic emergency		0.046	0.042	1.268
	Age	—	0.003	0.077	0.938
	Financial situation	—	−0.193	−5.741	<0.001 ^∗∗^
	Education	—	0.100	2.795	0.005 ^∗∗^
	Marital status	—	−0.016	−0.451	0.652
	Previous mood disorder diagnosis	—	0.208	6.259	<0.001 ^∗∗^
	Type of birth	—	−0.019	−0.520	0.603
	Type of pregnancy	—	−0.024	−0.662	0.508
	Child’s condition after birth	—	−0.009	−0.251	0.802
	Child’s birth complications		0.191	5.381	<0.001 ^∗∗^
	Birth weight		−0.122	−2.621	0.009 ^∗∗^
		△*F* (*df*1, *df*2)	13.906 ^∗∗^ (10, 782)		
		Total *R* ^2^/Adj. *R* ^2^	0.162/0.148		

*Note:* Ordinal variables having a minimum of five levels were treated as quantitative variables in the analysis. Ordinal variables having fewer than five levels were recorded into dummy variables: 1. preterm; birth during the COVID‐19 state of epidemic emergency: 1—yes, 0 – no; financial situation: 1—very bad, 2—bad, 3—average, 4—good, 5—very good; education: 1—basic education, 2—lower secondary school, 3—vocational certificate, 4—graduated from a secondary school, 5—postgraduate higher degree education; marital status: 1—married, 0—partnered, single parent or other; previous mood disorder diagnosed: 1—yes, 0—no; type of birth: 1—vaginal, 2—caesarean section; child’s condition after birth: 0—good, 1—hypoxia, asphyxia or no signs of life; type of pregnancy: 1—at‐risk, 2—low‐risk; child’s birth complications: 1—yes, 0—no.

^∗^ *p* ≤ 0.05.

^∗∗^ *p* ≤ 0.01.

The regression analysis (Table 7) for anxiety symptoms showed that the independent variables explained a total of 10.1% of the variance (*F*_(13, 782)_ = 6.72, *p*  < 0.001). Significant independent variables in Step 3 were the presence of COVID‐19 pandemic condition – the latter at a tendency level, financial situation, educational level, previous maternal mental health (showing that mothers who gave birth during the pandemic, with a worse financial situation, higher educational level and a prior diagnosis of a mood disorder, scored higher in anxiety symptoms) and a child’s physical health (showing that mothers of children born with medical complications and a lower birth weight scored higher in anxiety symptoms).

**Table 7:** Results of hierarchical regression analyses in which the degree of prematurity, giving birth during or after the COVID‐19 pandemic and selected aspects of socio‐demographic factors, maternal mental health prior to pregnancy, a child’s physical health and childbirth related factors were regressed upon anxiety symptoms .

Step	Predictor variable		Anxiety symptoms		
			Beta	*t*	*p*
1	Preterm group	Moderate (ref. very)	−0.091	−2.137	0.033 ^∗^
		Late (ref. very)	−0.072	−1.684	0.093^a^
*R* ^2^	0.006	△*F* (*df*1, *df*2)	2.46^a^ (2, 793)		
2	Preterm group	Moderate (ref. very)	−0.086	−2.005	0.045 ^∗^
		Late (ref. very)	−0.064	−1.496	0.135
	Birth during the COVID‐19 state of epidemic emergency		0.066	1.866	0.062^a^
*R* ^2^	0.011	△*F* (*df*1, *df*2)	3.484^a^ (1, 792)	—	—
3	Preterm group	Moderate (ref. very)	−0.016	−0.356	0.722
		Late (ref. very)	0.058	1.069	0.286
	Birth during the COVID‐19 state of epidemic emergency		0.063	1.813	0.070^a^
	Age	—	0.015	0.415	0.678
	Financial situation	—	−0.140	−4.012	<0.001 ^∗∗^
	Education	—	0.102	2.745	0.006 ^∗∗^
	Marital status	—	−0.056	−1.493	0.136
	Previous mood disorder diagnosis		0.179	5.187	<0.001 ^∗∗^
	Type of birth	—	−0.029	−0.775	0.439
	Type of pregnancy	—	−0.017	−0.453	0.651
	Child’s condition after birth	—	−0.005	−0.143	0.886
	Child’s birth complications		0.114	3.089	0.002 ^∗∗^
	Birth weight		−0.116	−2.408	0.016 ^∗^
		△*F* (*df*1, *df*2)	7.827 ^∗∗^ (10, 782)		
		Total *R* ^2^/Adj. *R* ^2^	0.101/0.086		

*Note:* Ordinal variables having a minimum of five levels were treated as quantitative variables in the analysis. Ordinal variables having fewer than five levels were recorded into dummy variables: 1. preterm; birth during the COVID‐19 state of epidemic emergency: 1—yes, 0 – no; financial situation: 1—very bad, 2—bad, 3—average, 4—good, 5—very good; Education: 1—basic education, 2—lower secondary school, 3—vocational certificate, 4—graduated from a secondary school, 5—postgraduate higher degree education; marital status: 1—married, 0—partnered, single parent or other; previous mood disorder diagnosed: 1—yes, 0 – no; type of birth: 1—vaginal, 2—caesarean section; child’s condition after birth: 0—good, 1—hypoxia, asphyxia or no signs of life; type of pregnancy: 1—at‐risk, 2—low‐risk; child’s birth complications: 1—yes, 0—no.

^a^ *p* ≤ 0.10 .

^∗^ *p* ≤ 0.05.

^∗∗^ *p* ≤ 0.01.

The regression analysis (Table 8) for anhedonia severity showed the independent variables explained a total of 11.6% of the variance (*F*_(13, 782)_ = 7.88, *p*  < 0.001). Significant independent variables in Step 3 were: financial situation, education, previous maternal mental health (showing that mothers with a worse financial situation, higher educational level and a prior diagnosis of a mood disorder scored higher in anhedonia symptoms) and a child’s physical health (showing that mothers of children born with medical complications and a lower birth weight at a tendency level scored higher in anhedonia symptoms).

**Table 8:** Results of hierarchical regression analyses in which the degree of prematurity, giving birth during or after the COVID‐19 pandemic and selected aspects of socio‐demographic factors, maternal mental health prior to pregnancy, a child’s physical health and childbirth related factors were regressed upon anhedonia symptoms .

Step	Predictor variable		Anhedonia severity		
			Beta	*t*	*p*
1	Preterm group	Moderate (ref. very)	−0.083	−1.945	0.052^a^
		Late (ref. very)	−0.079	−1.863	0.063^a^
*R* ^2^	0.006	△*F* (*df*1, *df*2)	2.333 (2, 793)		
2	Preterm group	Moderate (ref. very)	−0.082	−1.914	0.056^a^
		Late (ref. very)	−0.078	−1.816	0.070^a^
	Birth during the COVID‐19 state of epidemic emergency		0.013	0.373	0.710
*R* ^2^	0.006	△*F* (*df*1, *df*2)	0.139 (1, 792)	—	—
3	Preterm group	Moderate (ref. very)	−0.011	−0.247	0.805
	—	Late (ref. very)	0.035	0.659	0.510
	Birth during the COVID‐19 state of epidemic emergency		0.018	0.526	0.599
	Age	—	−0.037	−1.046	0.296
	Financial situation	—	−0.134	−3.895	<0.001 ^∗∗^
	Education	—	0.119	3.213	0.001 ^∗∗^
	Marital status	—	0.027	0.725	0.468
	Previous mood disorder diagnosis		0.144	4.203	<0.001 ^∗∗^
	Type of birth	—	−0.012	−0.319	0.750
	Type of pregnancy	—	−0.008	−0.213	0.831
	Child’s condition after birth	—	−0.027	−0.704	0.482
	Child’s birth complications		0.218	5.970	<0.001 ^∗∗^
	Birth weight		−0.083	−1.736	0.083^a^
		△*F* (*df*1, *df*2)	9.716 ^∗∗^ (10, 782)		
		Total *R* ^2^/Adj. *R* ^2^	0.116/0.101		

*Note:* Ordinal variables having a minimum of five levels were treated as quantitative variables in the analysis. Ordinal variables having fewer than five levels were recorded into dummy variables: 1. preterm; birth during the COVID‐19 state of epidemic emergency: 1—yes, 0—no; financial situation: 1—very bad, 2—bad, 3—average, 4—good, 5—very good; education: 1—basic education, 2—lower secondary school, 3—vocational certificate, 4—graduated from a secondary school, 5—postgraduate higher degree education; marital status: 1—married, 0—partnered, single parent or other; previous mood disorder diagnosed: 1—yes, 0—no; type of birth: 1—vaginal, 2—caesarean section; child’s condition after birth: 0—good, 1—hypoxia, asphyxia or no signs of life; type of pregnancy: 1—at‐risk, 2—low‐risk; child’s birth complications: 1—yes, 0—no.

^*a*^ *p* ≤ 0.10 .

^∗^ *p* ≤ 0.05.

^∗∗^ *p* ≤ 0.01.

The regression analysis (Table 9) for SHTs severity showed that the independent variables explained a total of only 4.4% of the variance (*F*_(13, 782)_ = 2.777, *p*  < 0.001). Significant independent variables in Step 3 were financial situation, education, previous maternal mental health (showing that mothers with a worse financial situation, higher educational level—a statistical trend, with a prior diagnosis of a mood disorder scored higher in SHTs symptoms).

**Table 9:** Results of hierarchical regression analyses in which the degree of prematurity, giving birth during or after the COVID‐19 pandemic and selected aspects of socio‐demographic factors, maternal mental health prior to pregnancy, a child’s physical health and childbirth related factors were regressed upon SHTs symptoms.

Step	Predictor variable		SHTs symptoms		
			Beta	*t*	*p*
1	Preterm group	Moderate (ref. very)	−0.038	−0.899	0.369
		Late (ref. very)	−0.011	−0.267	0.790
*R* ^2^	0.001	△*F* (*df*1, *df*2)	0.444 (2, 793)		
2	Preterm group	Moderate (ref. very)	−0.039	−0.901	0.368
		Late (ref. very)	−0.012	−0.273	0.785
	Birth during the COVID‐19 state of epidemic emergency		−0.003	−0.076	0.939
*R* ^2^	0.001	△*F* (*df*1, *df*2)	0.006 (1, 792)	—	—
3	Preterm group	Moderate (ref. very)	−0.043	−0.936	0.350
		Late (ref. very)	−0.027	−0.487	0.626
	Birth during the COVID‐19 state of epidemic emergency		−0.002	−0.065	0.973
	Age	—	0.001	0.034	0.973
	Financial situation	—	−0.100	−2.791	0.005 ^∗∗^
	Education	—	−0.065	−1.697	0.090 ^∗∗∗^
	Marital status	—	−0.019	−0.500	0.617
	Previous mood disorder diagnosis	—	0.150	4.226	<0.001 ^∗∗^
	Type of birth	—	0.032	0.828	0.408
	Type of pregnancy	—	−0.051	−1.311	0.190
	Child’s condition after birth	—	−0.039	−1.007	0.314
	Child’s birth complications		−0.009	−0.234	0.815
	Birth weight		0.035	0.709	0.479
		△*R* ^2^	3.518 ^∗∗^ (10, 782)		
		Total *R* ^2^/Adj. *R* ^2^	0.044/0.028		

*Note:* Ordinal variables having a minimum of five levels were treated as quantitative variables in the analysis. Ordinal variables having fewer than five levels were recorded into dummy variables: 1. preterm; birth during the COVID‐19 state of epidemic emergency: 1—yes, 0—no; financial situation: 1—very bad, 2—bad, 3—average, 4—good, 5—very good; education: 1—basic education, 2—lower secondary school, 3—vocational certificate, 4—graduated from a secondary school, 5—postgraduate higher degree education; marital status: 1—married, 0— partnered, single parent or other; previous mood disorder diagnosed: 1—yes, 0—no; type of birth: 1—vaginal, 2—caesarean section; child’s condition after birth: 0—good, 1—hypoxia, asphyxia or no signs of life; type of pregnancy: 1—at‐risk, 2—low‐risk; child’s birth complications: 1—yes, 0—no.

^∗^ *p* ≤ 0.05.

^∗∗^ *p* ≤ 0.01.

^∗∗∗^ *p* ≤ 0.10 .

## 4. Discussion

This study aimed to assess the impact of a premature birth during and after the COVID‐19 pandemic on the severity of maternal symptoms of depression, anxiety, anhedonia and SHTs. To the best of our knowledge, this is the first study to compare the prevalence of these symptoms during and after the pandemic among mothers of preterm children. Additionally, we aimed to investigate differences in the severity of depressive, anhedonia, anxiety and SHTs symptoms among mothers who gave birth very preterm, moderately preterm, and late preterm. Moreover, we explored socio‐demographic, psychological, birth‐related and COVID‐19 pandemic‐related factors associated with increased scores of these symptoms among mothers of preterm children.

Our findings did not confirm that giving birth during the pandemic impacted the incidence of PPD—as measured by depressive, anhedonia and SHT symptoms (both mean scores and cut‐off thresholds)—among mothers of preterm children. This aligns with studies from Italy [33], Estonia [40], and Chile [34] but contradicts one from Belgium that reported a significantly higher proportion of mothers exceeding the cut‐off point for possible clinical depression during the pandemic [37]. However, we found that pandemic conditions were associated with increased anxiety levels (a statistical trend) compared with postpandemic conditions. This result is in line with Brasseler et al.’s [39] study, which showed that PTB correlated with both depressive symptoms and heightened COVID‐19 anxiety and generalised anxiety. We found that, regardless of the state of epidemic emergency or restrictions, mothers who gave birth to very preterm children consistently exhibited higher depressive symptoms, as compared with mothers of moderately or late preterm children, but did not differ in anxiety, anhedonia and SHTs symptoms. Children born very preterm face a higher risk of neurodevelopmental and social–emotional challenges [1]. They require a longer and stressful hospitalisation in the NICU and experience more medical complications than children born moderately or late preterm [4, 5]. These circumstances are more stressful or even traumatic for parents, especially mothers, as the children’s physical symptoms which result from the PTB can persist till the preschool age [4, 26, 65]. However, they were associated with maternal probable clinical PPD, anxiety and SHTs (as measured with cut‐off points), pointing to only subtle differences in the levels of depressive symptoms between the mothers who gave birth to preterm, moderately preterm and late preterm babies. A similar finding was reported by Girchenko et al. [49]. In our study, 7% of mothers had an EPDS result indicating a clinical score for PPD, 10%—for anxiety and 3% experienced SHTs. These numbers are in line with our previous studies focusing on PPD, anxiety and SHTs [14, 66] in a general Polish postpartum population.

Moreover, we found that the common significant predictors of PPD and anhedonia severity among mothers of preterm infants included a worse financial situation, a higher educational level, a prior diagnosis of a mood disorder, the child’s poorer physical health (i.e. presence of medical complications) and lower birth weight—the latter, for anhedonia, on a tendency level only. For anxiety, the significant predictors included all the above‐mentioned variables, and additionally, giving birth during the COVID‐19 pandemic, which may be associated with a high comorbidity of PPD and anxiety symptoms [67]. Mothers of preterm infants often experience heightened postnatal distress and anxiety [8–10]. The anxiety could have been further intensified by the COVID‐19 pandemic. We assume that the increase of anxiety symptoms could be associated with the fact that the potentially dangerous and unknown virus could serve as an additional risk factor for the health, development and survival of children who are already frequently affected by respiratory problems and disorders.

According to van der Zee‐van et al.[68], anxiety, anhedonia and depression have both shared and separate risk factors. This was confirmed in our study, as the pandemic situation impacted only the anxiety level, while other mother—and child‐related factors were common. The history of psychiatric symptoms was also identified as a predictor of PPD by Girchenko et al. [49] and Dennis et al. [69]. Moreover, a family social risk was documented as a predictor of distress among mothers of preterm children [70], and a worse financial situation: being unemployed was shown to be associated with anxiety state and trait in a smaller Alexopoudoulu et al.’s study [71]. In Helle et al. ’s [27] study the most relevant risk factor for maternal mental health problems was very low birthweight, which is also in line with our results. Furthermore, our model explained only 4.6% of the variance in the severity of SHTs. The only statistically significant predictors were worse financial situation, higher educational level (a statistical trend) and a prior mood disorder diagnosis. Similarly, a low socioeconomic status and a prior psychiatric diagnosis were also reported as predictors of SHTs by Palladino et al. [72], who conducted their study in the general population of postpartum women, not only one of PTB mothers.

The recent systematic review and meta‐analysis by Nguyen et al. [67] focusing on anxiety symptoms does not identify associated risk factors, unlike in the case of depression, which highlights a variety of risk factors associated with geographic regions. The models in our study show the predictive role of worse financial situation for depressive and anxiety symptoms with privileged education situation as risk factors.

Although the predictive value of our models is limited, we believe our study has several practical implications. Targeting maternal stress and mental health symptoms is possible during the baby’s stay in the hospital, especially when additional risk factors are known. According to the literature, intervention programmes that aim to reduce stress in parents of preterm infants and improve their competencies are quite common in NICUs and effective in lowering maternal PPD and anxiety risk [73, 74]. Based on our previous studies [47] and following the above‐cited studies, we hypothesised that stress‐coping workshops/interventions may be more accessible and beneficial for parents of preterm children than direct PPD prevention or treatment interventions, which are effective, but may be also stigmatising and not perceived as appropriate by parents. Moreover, online interventions are associated with a decrease in stress and maternal mental health symptoms. For example, in Taiwan, a video visitation offered during the COVID‐19‐related restrictions reduced maternal depression in mothers of preterm infants [38].

## 5. Strengths and Limitations

The study has several strengths. First, it used data derived from a state‐led and state‐funded PPD prevention programme coordinated by the Ministry of Health, reflecting real‐world conditions. This kind of study design enhances the ecological validity of the findings and increases their relevance for clinical practice. Moreover, all new mothers in northern Poland were approached for screening and assessed by certified medical professionals during routine postpartum care. This approach allowed us to include mothers who gave birth during pandemic‐related restrictions, thereby minimising selection bias. Another key strength is the relatively large sample size, particularly given the specific and vulnerable population of mothers of preterm infants. This sizeable sample also enabled a detailed differentiation of PTBs, allowing for subgroup analyses of very preterm, moderate preterm and late preterm infants. Finally, the use of a comprehensive multivariate predictor model, not limited to the COVID‑19–related factors, constitutes an additional strength of the study.

However, only mothers participated in the midwife‐led screening. The lack of fathers as respondents in our study is a limitation, as fathers experience a high level of stress and experience a lack of control [75]. Moreover, studies emphasise the need for including early routine screening and parenting support for fathers in perinatal health services [76]. We also did not have access to detailed data about the history of maternal mental and physical health and possible complications during pregnancy, such as depression during pregnancy, placenta previa, chorioamnionitis and premature rupture of membranes. All these factors could have modified the effects of PTB on maternal PPD. However, we could only identify a general group of at‐risk mothers, without having more specific information about their medical status. Similarly, we did not have access to the detailed data about a child’s medical complications. As we know PTB can cause various medical complications [77], so the impact of this factor may differ depending on the level of complexity and severity. Nevertheless, the study addresses clinically meaningful issues that are highly relevant to parents and practitioners and may enhance preventive strategies and psychological support in perinatal care. Furthermore, our study was not a longitudinal study. Comparisons were made between two different groups of women: one group was assessed during the pandemic, and another—after the restrictions ended. Such an analysis is limited and may have affected the results we obtained. Our earlier study [14] showed a significant discrepancy between an online and midwife‐led EPDS screening, with lower scores observed in the midwife‐led screening. We attributed these differences to a potential fear of stigmatisation and lack of anonymity, which could have influenced our results also in the present study.

## 6. Conclusions

In conclusion, our findings suggest that:

1. A very PTB is associated with an increase in depressive symptoms, regardless of the fact that the delivery occurred during the pandemic restrictions.
2. The COVID‐19 pandemic was associated with an increase in anxiety symptoms among PTB mothers.
3. The predictors for the severity of PPD, anxiety and anhedonia were: worse financial situation, a higher educational level, a prior maternal mood disorder diagnosis and a child’s physical health (a lower birth weight and the presence of medical complications). Additionally, the severity of anxiety symptoms was predicted by giving birth during the COVID‐19 pandemic.

## Funding

Analysed data were collected within the framework of the project. The Next Stop: Mum, which is a part of the National Health Policy Program of the Ministry of Health, entitled The Program of Education and Prevention of Postpartum Depression (Number POWR.05.01.00‐IP.05‐00‐006/18). The project is implemented on the basis of a contract with the Ministry of Health and is a part of the Operational Programme: Knowledge Education Development 2014–2020, co‐financed from the European Social Fund.

## Disclosure

Analysed data were collected within the framework of the project. The Next Stop: Mum, which is a part of the National Health Policy Program of the Ministry of Health, entitled The Program of Education and Prevention of Postpartum Depression (Number PPOWR.05.01.00‐IP.05‐00‐006/18).

## Conflicts of Interest

The authors declare no conflicts of interest.

## Endnotes

1

## Supporting Information

Additional supporting information can be found online in the Supporting Information section.

## Supporting information


**Supporting Information** Semi‐structured interview questionnaire developed by the first author. The instrument includes items assessing socio‐economic status (SES), marital status, place of residence, maternal and neonatal peripartum health indicators (e.g., birth weight, gestational age, length of NICU stay), and the child’s health complications. Data were collected by a trained midwife during a semi‐structured interview based on this questionnaire.

## Data Availability

The data that support the findings of this study are available from the corresponding author upon reasonable request.

## Footnotes

1
^1^Authors [78] reported initial evaluations of the PPD screening programme made by 150 randomly selected mothers. Authors evaluated the PPD Prevention Strategy in Poland after the first 2 years of its implementation [79]. The authors [14] examined the cooccurrence of PPD and anxiety disorder. The authors [66] assessed the prevalence of self‐harm thoughts among mothers in the postpartum period.
