# Supplementary material for: Postpartum Mental Health Problems in the Mothers of Very, Moderately and Late Preterm Infants During and After COVID–19 Pandemic: A Cross‐Sectional Comparison Study and Possible Risk Factors
Source: Depress Anxiety. 2026 May 14;2026:5236584. doi: 10.1155/da/5236584 (PMC13176785; doi:10.1155/da/5236584)
Supplement: Supplementary file 1 — Supporting Information Semi‐structured interview questionnaire developed by the first author. The instrument includes items assessing socio‐economic status (SES), marital status, place of residence, maternal and neonatal peripartum health indicators (e.g., birth weight, gestational age, length of NICU stay), and the child’s health complications. Data were collected by a trained midwife during a semi‐structured interview based on this questionnaire. [file DA-2026-5236584-s001.pdf]

SOCIO-DEMOGRAPHIC QUESTIONNAIRE FOR PARTICIPANTS WITHIN THE "PRZYSTANEK MAMA" PROJECT

Please join in completing the Edinburgh Postnatal Depression Scale. Date: ....

Name (mother):	Identification number of the midwife/doctor
Mother's Contact Phone:	Child's Gender:
Mother's Age (in years):	Number of children:
Child's Date of Birth (date of birth of the youngest child / DD-MM-YYYY):	
Marital Status (underline):<br>marriage / civil partnership / cohabitation / informal / single parent / other	
Education (underline):<br>primary / lower secondary / basic vocational / secondary / higher	
Place of Residence (underline):<br>village/town up to 50 thousand inhabitants/town up to 100 thousand inhabitants/town up to 250 thousand inhabitants/city over 250 thousand inhabitants	
How do you rate your financial situation (underline, please):<br>very good / good / average / poor / very poor	
Have you ever suffered from (underline, please):<br>depression / bipolar disorder / other mood disorders / none of the above	
Week of pregnancy when childbirth occurred:	
**Birth weight (grams):**	
Type of childbirth: ☐ physiological ☐ cesarean section	
Type of pregnancy (in relation to it's course): ☐ at risk ☐ not at risk	
Child's condition after birth: good / hypoxia / asphyxia / no signs of life	
Apgar score: initial: final:	
Congenital defects and genetic syndromes in the child<br>(if any, enter the diagnosis):	
Duration of the child's stay in the intensive care unit / premature baby ward (in days):	
Duration of stay in the incubator (in days):	
Were there any complications in the child after birth?<br>no / yes, Please describe:	
Did you experience any complications after childbirth?<br>no / yes Please describe:	

Project No. POWR.05.01.00–00–0023 / 18–00 Pt. "Przystanek MAMA" is co-financed by the European Social Fund under Measures 5.1 Preventive Programs of the Operational Program "Knowledge, Education, and Development" for 2014–2020.
